# Supplementary material for: Epigenetic modification and characterization of the MGMT promoter region using CRISPRoff in glioblastoma cells
Source: Front Oncol. 2024 Jan 31;14:1342114. doi: 10.3389/fonc.2024.1342114 (PMC10864556; doi:10.3389/fonc.2024.1342114)
Supplement: Supplementary file 1 [file Image_1.pdf]

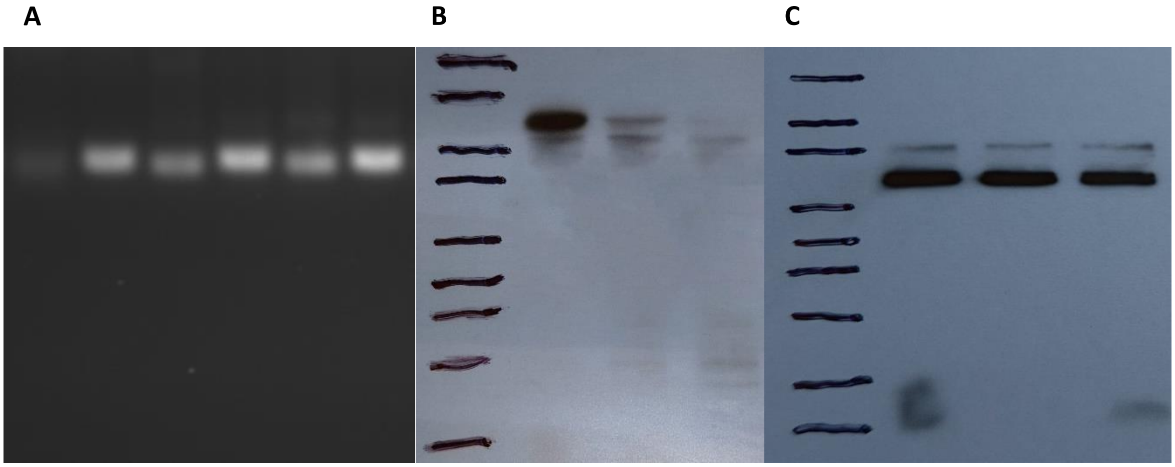

Supplementary Figure 1: Original immunoblots and agarose gels. (A) Agarose gel of methylation-specific PCR products to quantify MGMT promoter methylation of the three T-325 cell lines (B) MGMT protein levels in T-325 cells quantified by immunoblot. (C) Actin protein levels in T-325 cells quantified by immunoblot.
